# Alpha-COPI Coatomer Protein Is Required for Rough Endoplasmic Reticulum Whorl Formation in Mosquito Midgut Epithelial Cells

**DOI:** 10.1371/journal.pone.0018150

**Published:** 2011-03-31

**Authors:** Guoli Zhou, Jun Isoe, W. Antony Day, Roger L. Miesfeld

**Affiliations:** 1 Department of Chemistry and Biochemistry, The University of Arizona, Tucson, Arizona, United States of America; 2 Arizona Research Labs, The University of Arizona, Tucson, Arizona, United States of America; University of South Florida College of Medicine, United States of America

## Abstract

**Background:**

One of the early events in midgut epithelial cells of *Aedes aegypti* mosquitoes is the dynamic reorganization of rough endoplasmic reticulum (RER) whorl structures coincident with the onset of blood meal digestion. Based on our previous studies showing that feeding on an amino acid meal induces TOR signaling in *Ae. aegypti*, we used proteomics and RNAi to functionally identify midgut epithelial cell proteins that contribute to RER whorl formation.

**Methodology/Principal Findings:**

Adult female *Ae. aegypti* mosquitoes were maintained on sugar alone (unfed), or fed an amino acid meal, and then midgut epithelial cells were analyzed by electron microscopy and protein biochemistry. The size and number of RER whorls in midgut epithelial cells were found to decrease significantly after feeding, and several KDEL-containing proteins were shown to have altered expression levels. LC-MS/MS mass spectrometry was used to analyze midgut microsomal proteins isolated from unfed and amino acid fed mosquitoes, and of the 127 proteins identified, 8 were chosen as candidate whorl forming proteins. Three candidate proteins were COPI coatomer subunits (alpha, beta, beta'), all of which appeared to be present at higher levels in microsomal fractions from unfed mosquitoes. Using RNAi to knockdown alpha-COPI expression, electron microscopy revealed that both the size and number of RER whorls were dramatically reduced in unfed mosquitoes, and moreover, that extended regions of swollen RER were prevalent in fed mosquitoes. Lastly, while a deficiency in alpha-COPI had no effect on early trypsin protein synthesis or secretion 3 hr post blood meal (PBM), expression of late phase proteases at 24 hr PBM was completely blocked.

**Conclusions:**

alpha-COPI was found to be required for the formation of RER whorls in midgut epithelial cells of unfed *Aa. aegypti* mosquitoes, as well as for the expression of late phase midgut proteases.

## Introduction

Blood meal digestion takes place in the midgut of hematophagous arthropods and provides the source of nutrients for vitellogenesis and oogenesis, as well as the entry point for pathogen transmission [1]. Mosquito midgut epithelial cell ultrastructures have previously been characterized by electron microscopy in several species, including *Aedes aegypti* [2], [3], [4], [5], *Anopheles gambiae* [6], *An. darlingi* [7], *Culex quinquefasciatus* [8], and *Cx. tarsalis* [9]. One of the most striking findings from these studies was that the rough endoplasmic reticulum (RER) in midgut epithelial cells of adult female *Aedes* and *Anopheles* mosquitoes is organized into large whorl-like structures prior to blood feeding. With the onset of feeding, the RER whorls undergo a dramatic reorganization, presumably to facilitate the synthesis and secretion of digestive proteases and components of the peritrophic matrix [2], [3], [5], [7], [10], [11]. Interestingly, male mosquitoes, which do not blood feed, lack RER whorls in their midgut epithelial cells [12]. The identity and functional contribution of protein components localized to mosquito midgut RER whorls have not been investigated.

ER whorls have been shown to form in vertebrate cells exposed to inducers of the ER stress response [10], [13], and as a result of pathological conditions [14]. Whorl-like smooth ER structures have also been shown to form in mammalian tissue culture cells that over express transfected ER resident proteins or fluorescent protein gene fusions containing heterologous transmembrane domains [15], [16], [17]. Immunofluorescent staining has been used in these transfection experiments to show that weak protein-protein interactions between the cytoplasmic domains of highly abundant ER imbedded proteins are sufficient to induce membrane stacking and whorl formation. The absence of some ER proteins can also induce whorl formation, suggesting that mechanisms are in place to prevent ER whorls from forming in most cell types [18].

Plasma free amino acids and protein-bound amino acids in vertebrate blood provide the raw materials for energy conversion and reproduction [19], [20], [21], and also function to initiate signaling through the target of rapamycin (TOR) signal transduction pathway [22], [23]. Moreover, an artificial amino acid meal is sufficient to induce translation of early trypsin mRNA using the TOR signaling pathway in midgut epithelial cells, however amino acids alone do not induce the transcription and translation of the late phase trypsin protein [24]. It is possible that amino acid signaling through the TOR pathway also stimulates RER reorganization in mosquito midgut epithelial cells.

In order to investigate mechanisms controlling RER whorl unwinding and the initiation of blood meal digestion in female *Ae. aegypti* mosquitoes, we performed proteomic studies using mass spectrometry and identified midgut proteins associated with microsomal fractions in unfed and amino acid fed mosquitoes. The 139 kDa alpha subunit of the COPI coatomer complex was selected as one of eight candidate RER whorl-forming proteins based on bioinformatic analyses. We used RNAi-mediated knockdown to determine if loss of alpha-COPI expression had any effect on RER whorl formation in unfed mosquitoes or blood meal digestion in fed mosquitoes. Our data show that alpha-COPI functions are indeed required for RER whorl formation in unfed mosquitoes, and moreover, that a deficiency in alpha-COPI blocked the expression of three late phase midgut proteases in blood fed mosquitoes (AaSPVI, AaSPVII, AaLT). Interestingly, the early phase of blood meal digestion, which is characterized by the synthesis and secretion of the early trypsin protease (AaET), was not dependent on alpha-COPI expression.

## Results

### Amino acid feeding induces RER whorl unwinding

**Figure 1:**
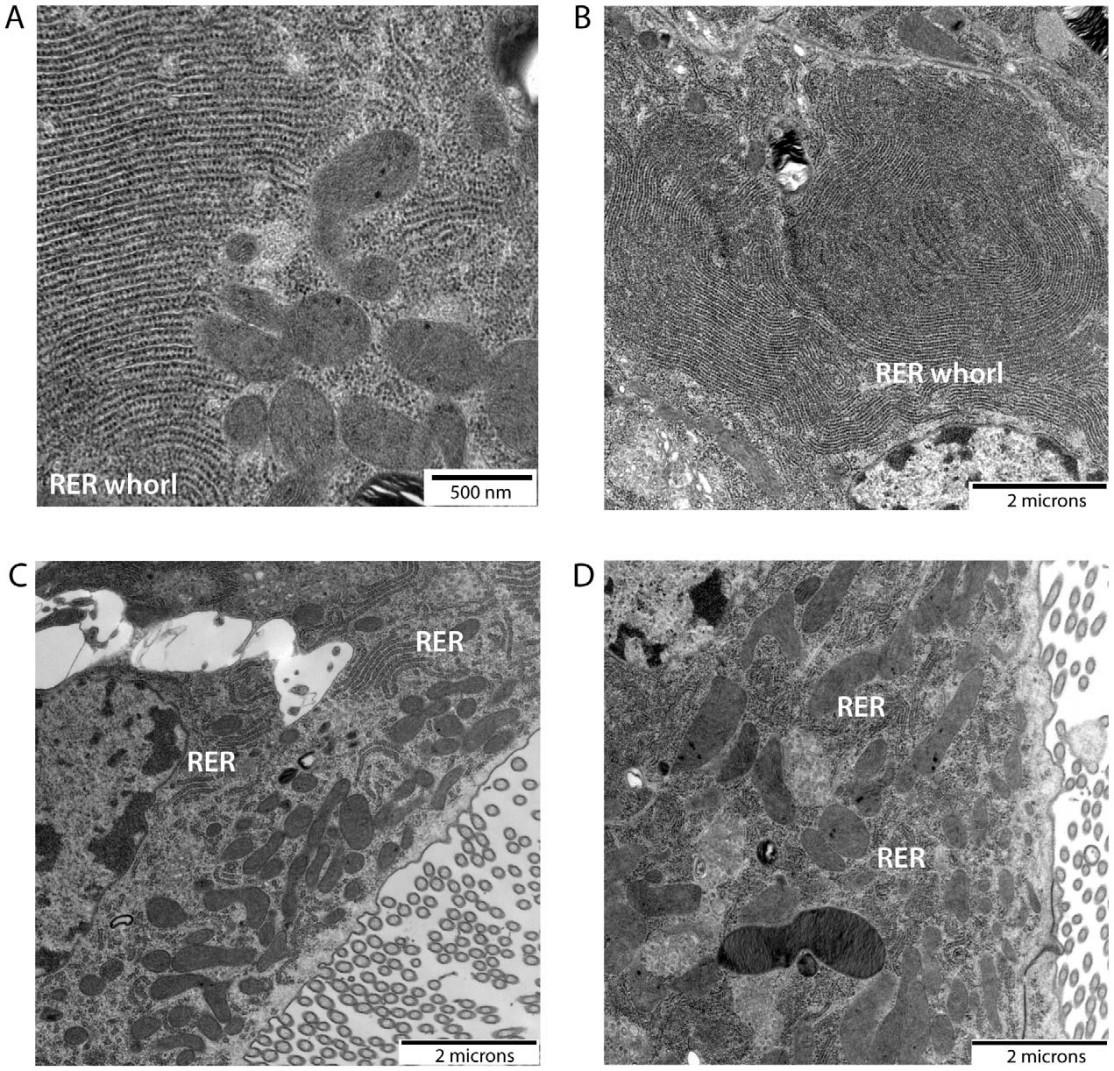
Amino acid feeding is sufficient to induce RER whorl unwinding. Three-day-old *Ae. aegypti* females were kept unfed (sugar fed), or fed an amino acid meal for 30 min., and then sacrificed and dissected 30 min. or 120 min. later. The dissected midguts were fixed for electron microscopy preparation as described in Materials and Methods and representative electron micrographs of midgut sections are shown here. A) Unfed mosquito (25,000× magnification). B) Unfed mosquito (8,800× magnification). C) Amino acid fed mosquito dissected at 30 min. post-feeding (8,800× magnification). D) Amino acid fed mosquito dissected at 120 min. post-feeding (8,800× magnification).

**Figure 2:**
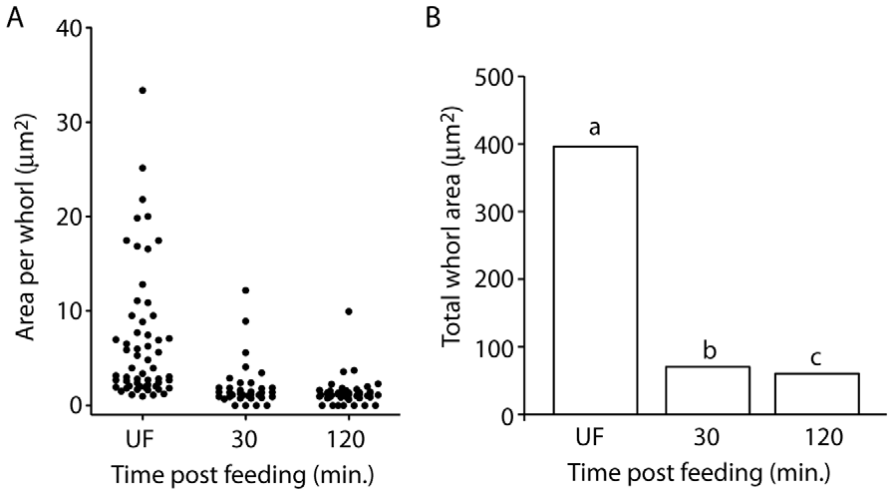
Quantitation of whorl size and number in unfed and amino acid fed mosquitoes. Whorls with a minimum of five stacked ER membranes were counted and the total area of each whorl was determined using NIH Image software. Data are shown for whorl size and number in 20 random EM fields covering 3335 µm^2^ of mosquito midgut epithelial cell area. A) Size and number of RER whorls in 20 random fields from unfed and amino acid fed mosquitoes after 30 min. and 120 min. B) Total whorl in the same sample as shown in A. Statistical analysis using Pearson Chi-square test revealed that the total whorl area in amino acid fed mosquitoes at both 30 min and 120 min post-feeding was significantly less than the total whorl area in unfed mosquitoes (bars with different letters signify significance at p<0.001 comparing 30 min and 120 min to unfed mosquitoes).

To determine if amino acids in the midgut lumen are sufficient to trigger RER whorl unwinding in midgut epithelial cells of *Ae. aegypti* mosquitoes, females were fed an artificial amino acid meal and dissected midguts were characterized for ultrastructural changes at the subcellular level using electron microscopy (EM). As seen in representative electron micrographs (Figure 1), large whorls of stacked RER membranes were observed in midgut epithelial cells from unfed (sugar only) mosquitoes, with some RER whorls containing >50 stacked membranes. The whorls appeared to consist entirely of tightly packed RER membranes, with no evidence of a core lipid droplet as seen in other organisms [25]. Quantitative measurements of whorl size in 20 randomly chosen EM fields revealed that the RER whorls ranged in size from ∼5–35 µm^2^ in unfed mosquitoes (Figure 2). However, following a 30 minute amino acid feeding period, and another 30 minutes of recovery, midgut epithelial cells were found to contain significantly fewer RER whorls of >5 membrane stacks (p<0.001) (Figure 2). Moreover, most of the RER contained in the midgut epithelial cells of amino acid fed mosquitoes was arranged in short linear stacks (Figure 1C). Quantitative measurements of RER whorl size and number in midgut epithelial cells following a recovery period of 120 minutes after amino acid feeding gave similar results, suggesting that RER whorl unwinding is rapid and complete by 30 minutes post feeding (Figures 1D and 2).

### Identification of candidate RER whorl-associated proteins

**Figure 3:**
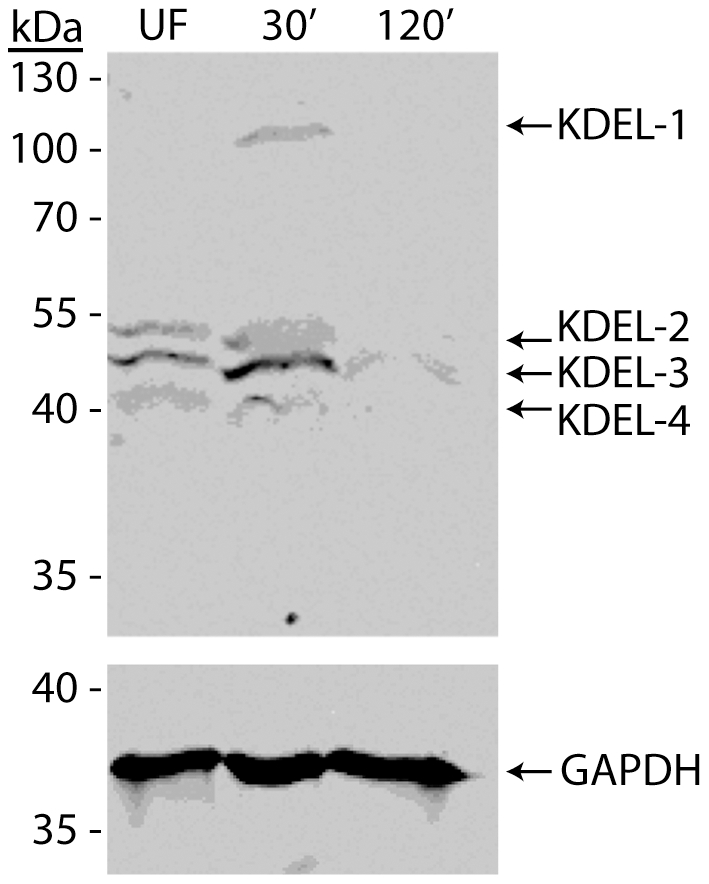
Western blot analysis using a KDEL-specific antibody shows differential expression of four ER resident midgut proteins in response to amino acid feeding in *Ae. aegypti* mosquitoes. Total midgut protein extracts were prepared from three day old female mosquitoes that were unfed, or amino acid fed and dissected at 30 min. or 120 min. post-feeding. Proteins were resolved by 12% SDS-PAGE gel and Western blotted as described in Materials and Methods. The four unique protein bands were labeled KDEL-1 through KDEL-4 to denote their relative molecular weight, with KDEL-1 being the largest protein. Western blotting with an antibody that recognizes the ubiquitously expressed glyceraldehyde-3P dehydrogenase protein (GAPDH) was performed to control for equal protein loading.

Reorganization of the ER in midgut epithelial cells of amino acid fed mosquitoes may be associated with differential abundance of KDEL-containing ER resident proteins, which could provide a clue as to possible whorl-enriched proteins. To test this possibility, we prepared total protein extracts from midguts of unfed and amino acid fed (30 min and 120 min post feeding) mosquitoes, and analyzed the extracts by Western blotting using a KDEL-specific antibody as shown in Figure 3. Four distinct protein bands were observed, two of which appeared to decrease in abundance after feeding (KDEL-2 and KDEL-4), relative to the internal control protein (GAPDH). The KDEL-1 protein was only present in the 30 min sample, whereas the KDEL-3 protein was present in all three protein samples. Since *Ae. aegypti* protein disulfide isomerase (PDI) has a predicted molecular mass of ∼56 kDa, and is a KDEL-containing ER protein known to be expressed in *Ae. aegypti* midgut cells [26], we analyzed these same protein extracts with a PDI antibody that recognizes *Drosophila melanogaster* PDI. Results from these experiments were negative (data not shown), however we could not rule out that antigenic differences between *Drosophila* and *Aedes* PDI proteins contributed to the negative result.

**Figure 4:**
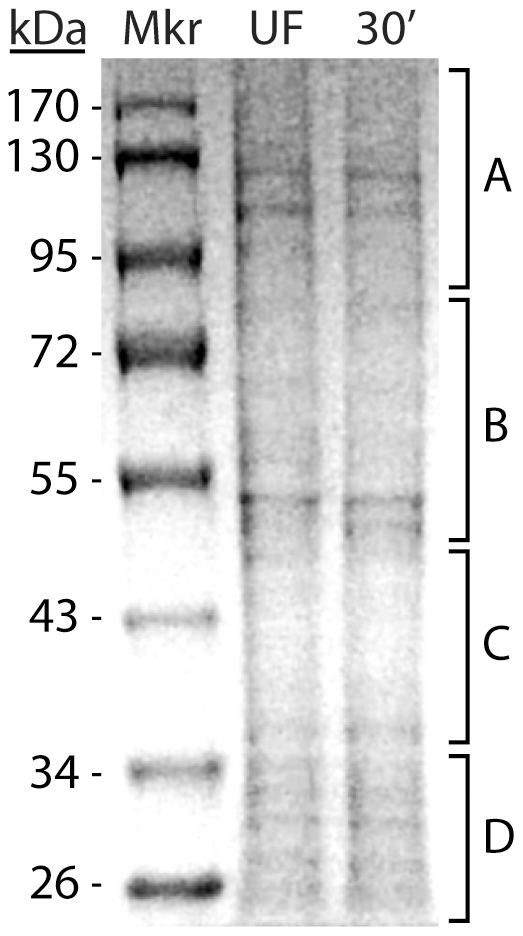
Isolation of microsomal proteins for LC-MS/MS analysis from unfed and amino acid fed *Ae. aegypti* mosquitoes. Three-day-old female mosquitoes were unfed or amino acid fed and dissected after 30 min. Microsomal protein samples were prepared as described in Materials and Methods and resolved on a 12% SDS-PAGE gel. Following protein staining, four equal slices of polyacrylamide gel were prepared from each lane (A, B, C, or D) and proteins were subjected to in-gel trypsin digestion prior to LC-MS/MS analysis. Note that minimal differences exist in the quality and quantity of abundant proteins in the two protein samples, suggesting that the 30 min. time point is prior to the onset of feeding-induced protein synthesis.

To more directly identify midgut proteins that might be contributing to whorl formation in unfed adult female mosquitoes, we isolated microsomal proteins from midgut tissues of unfed and amino acid fed mosquitoes (30 min post-feeding) using a modified procedure that minimized protease activity in the sample and enriched for RER associated proteins (see Materials and Methods). The protein samples were separated by molecular weight using SDS PAGE and four gel slices from each lane were processed for LC-MS/MS analysis and protein annotation (Figure 4). The SDS PAGE analysis revealed that intact proteins of up to ∼130 kDa were present in both samples, suggesting that protease activity was minimal in the midgut protein sample from fed mosquitoes. Moreover, no major differences were observed in the distribution of proteins isolated from the unfed or amino acid fed mosquitoes, indicating that the 30 min time point preceded feeding-induced protein synthesis.

Following in-gel trypsin digestion, each of the eight protein samples was analyzed by LC- MS/MS. Bioinformatic analysis of the mass spectrometry data led to the identification of 127 proteins using the *Ae. aegypti* genome database in a Sequest-based search (Table S1). Gene Ontology (GO) analysis revealed that ∼30% of the identified proteins could be assigned to microsomes (including ribosomal proteins and protein synthesis related proteins), ∼19% were mitochondrial proteins, ∼37% were cytoplasmic proteins, and ∼14% were from other cellular fractions, including the nucleus. Eight of the microsomal proteins that were present in the protein sample from unfed mosquitoes, were chosen as candidate whorl-associated proteins based on their known role in ER or Golgi processes (Table 1). Based on peptide frequency, the most often represented protein was SND1 (Staphylococcal nuclease domain-containing protein 1), which is also known as Tudor domain-containing protein 11 [27]. Although SND1 is assigned to the Golgi apparatus cellular component by GO analysis, its primary function is gene regulation and RNA processing [28]. The peptide frequency of SND1 was similar in protein samples from unfed and fed mosquitoes, even though EM analysis revealed that the majority of RER whorl unwinding had already occurred by 30 min post amino acid feeding (Figure 1C). This result suggests that SND1 is most likely not associated with whorl maintenance since its presence in microsomal fractions is independent of whorl formation.

**Table 1:** ER and Golgi associated proteins identified by mass spectrometry.

Protein name	Mol. Wt.	Unfed (#hits)	30' Fed (#hits)	Cellular component	Biological process	Accession number
alpha-COPI	139 kDa	7	0	COPI vesicle coat	Vesicle-mediated transport	XP_001663309
beta-COPI	107 kDa	8	4	COPI vesicle coat	Vesicle-mediated transport	XP_001649392
beta'-COPI	106 kDa	6	0	COPI vesicle coat	ER to Golgi vesicle-mediated transport	XP_001657383
SND1	103 kDa	22	26	Golgi apparatus	Transcription	XP_001654799
SRP68	70 kDa	2	0	Endoplasmic reticulum	Response to drug	XP_001660604
PAST-1	61 kDa	3	0	Coated pit, endocytic vesicle	Vesicle organization and biogenesis	XP_001654538
PDI	56 kDa	3	0	Endoplasmic reticulum	Cell redox homeostasis	XP_001649775
RACK-1	35 kDa	5	6	COPI vesicle coat	Golgi vesicle transport	XP_001863303

Eight proteins out of the 127 identified by mass spectrometry were shown by GO analysis to be associated with ER or Golgi membranes. Number of peptides identified by Sequest in protein samples analyzed my mass spectrometry from unfed and fed mosquitoes are shown. Cellular component and biological process descriptions are based on GO terms assigned to each protein.

Besides PDI, we also identified three of the seven known coatomer subunits of the COPI vesicle transport system. As shown in Table 1, more peptides corresponding to the alpha-COPI, beta-COPI, beta'-COPI, subunits were identified in the microsomal fractions isolated from unfed mosquitoes than fed mosquitoes (Table 1), indicating that endosomal membranes in these fractions were depleted of COPI subunits. Since COPI subunits are soluble proteins that transiently cycle between vesicle membrane bound and unbound forms as a function of interactions with Arf proteins [29], [30], the observed differential abundance of COPI subunits in the two midgut microsomal fractions suggests that they could be associated with RER whorls. We tested this idea by knocking down alpha-COPI expression using RNAi to determine if this COPI coatomer subunit is required for RER whorl structures in mosquito midgut epithelial cells.

### alpha-COPI expression is required for RER whorl formation in midgut epithelial cells

**Figure 5:**
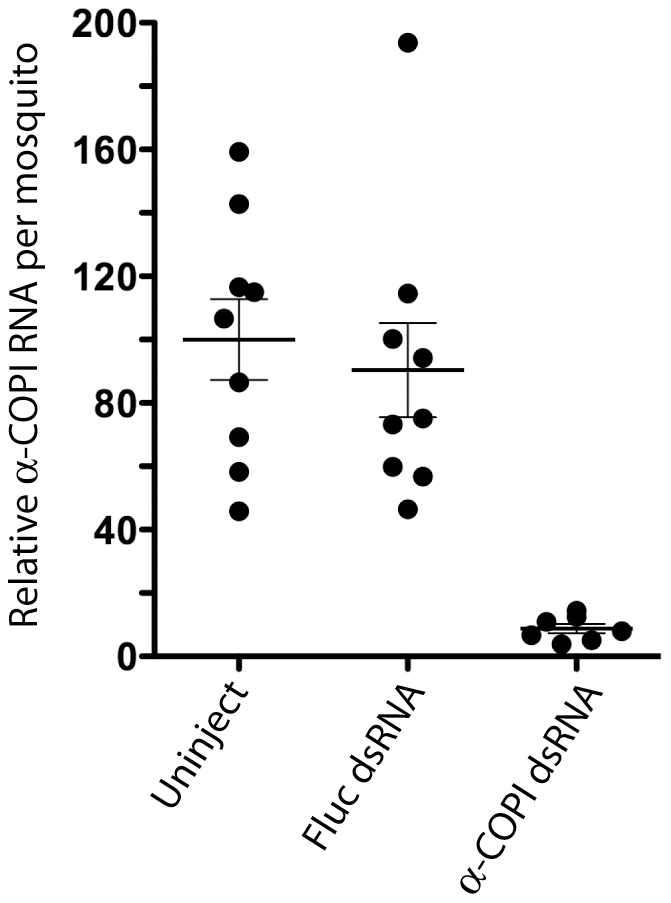
QRT-PCR analysis of alpha-COPI transcript levels in midgut tissue of individual *Ae. aegypti* mosquitoes that were either uninjected, or injected with dsRNA targeted against the control Fluc gene or the alpha-COPI gene. Transcript analysis revealed that 100% of the alpha-COPI dsRNA injected mosquitoes had <10% the level of alpha-COPI transcripts present in uninjected or Fluc dsRNA injected mosquitoes.

In order to test the role of alpha-COPI in RER whorl formation, we used an efficient dsRNA based RNAi protocol we previously developed for knocking down expression of abundant midgut proteases in *Ae. aegypti* [31]. As shown in Figure 5, the mean level of alpha-COPI transcripts in midguts of individual dsRNA-injected mosquitoes was >90% lower than the mean level in both uninjected mosquitoes and mosquitoes that had been injected with a control dsRNA from the firefly luciferase gene (Fluc). Since 100% of the alpha-COPI dsRNA injected mosquitoes we analyzed showed a similar decrease in alpha-COPI transcript levels (Figure 5), we used this dsRNA injection protocol to knock down alpha-COPI expression in mosquitoes that were subsequently maintained on sugar alone (unfed), or fed an amino acid meal and sacrificed 120 min post-feeding.

**Figure 6:**
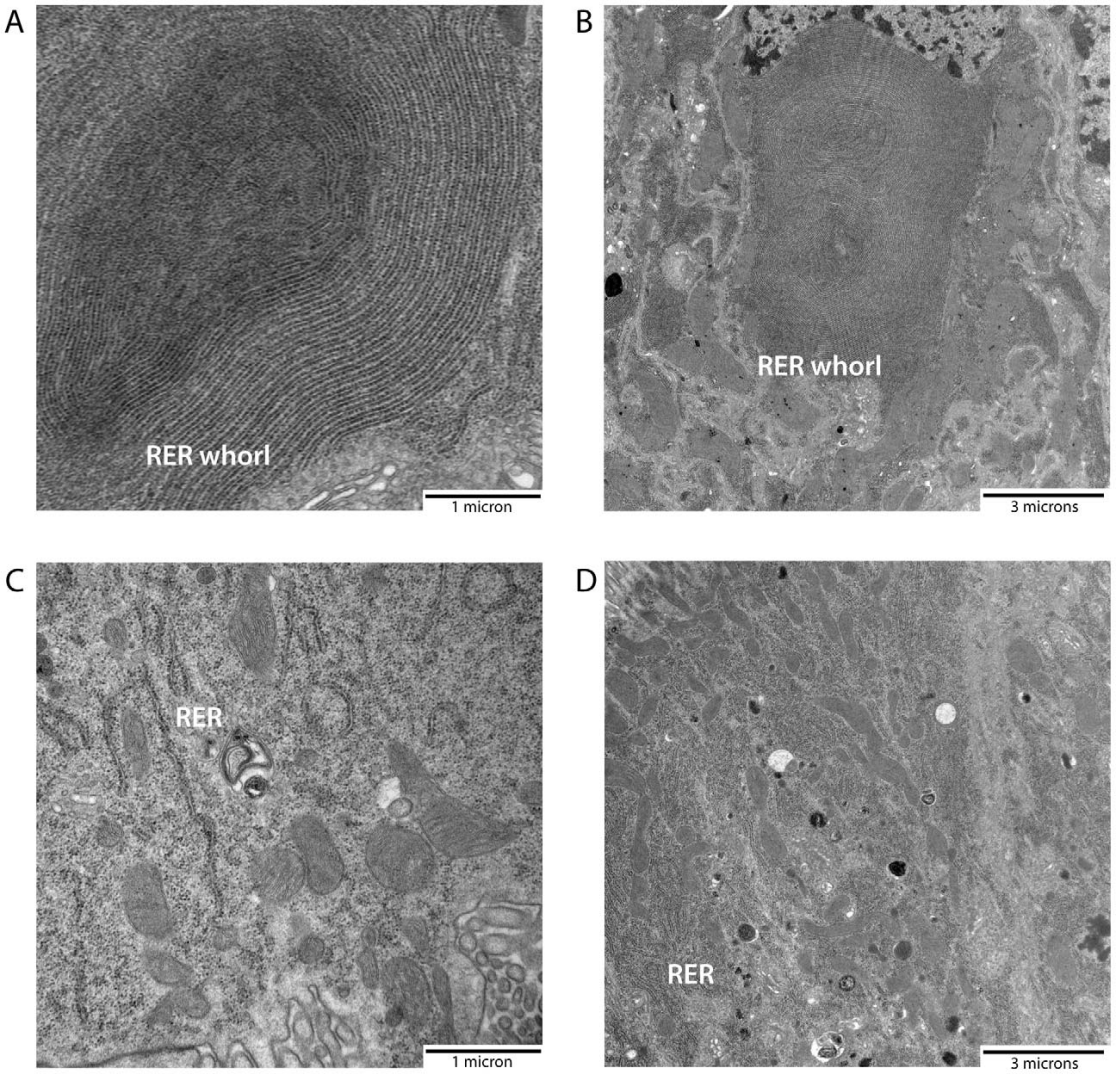
Knock down of alpha-COPI expression is associated with disorganization of RER whorls in unfed mosquitoes. Representative electron micrographs are presented here. A) Unfed Fluc dsRNA injected mosquito (25,000× magnification). B) Unfed Fluc dsRNA injected mosquito (8,800× magnification). C) Unfed alpha-COPI dsRNA injected mosquito (25,000× magnification). D) Unfed alpha-COPI dsRNA injected mosquito (8,800× magnification).

**Figure 7:**
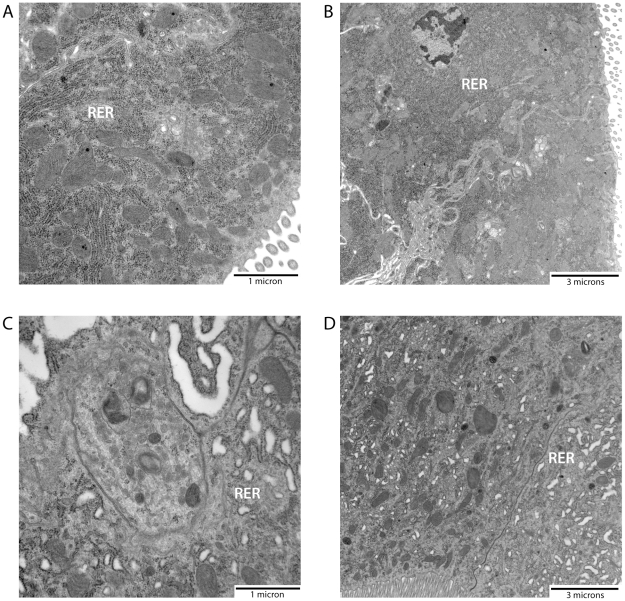
Knock down of alpha-COPI expression is associated with disorganization of endosomal structures and appearance of extended regions of swollen RER in amino acid fed mosquitoes. Representative electron micrographs are presented here. A) Fluc dsRNA injected mosquito dissected 120 min. after amino acid feeding (25,000× magnification). B) Fluc dsRNA injected mosquito dissected 120 min. after amino acid feeding (8,800× magnification). C) alpha-COPI dsRNA injected mosquito dissected 120 min. after amino acid feeding (25,000× magnification). D) alpha-COPI dsRNA injected mosquito dissected 120 min. after amino acid feeding (8,800× magnification).

**Figure 8:**
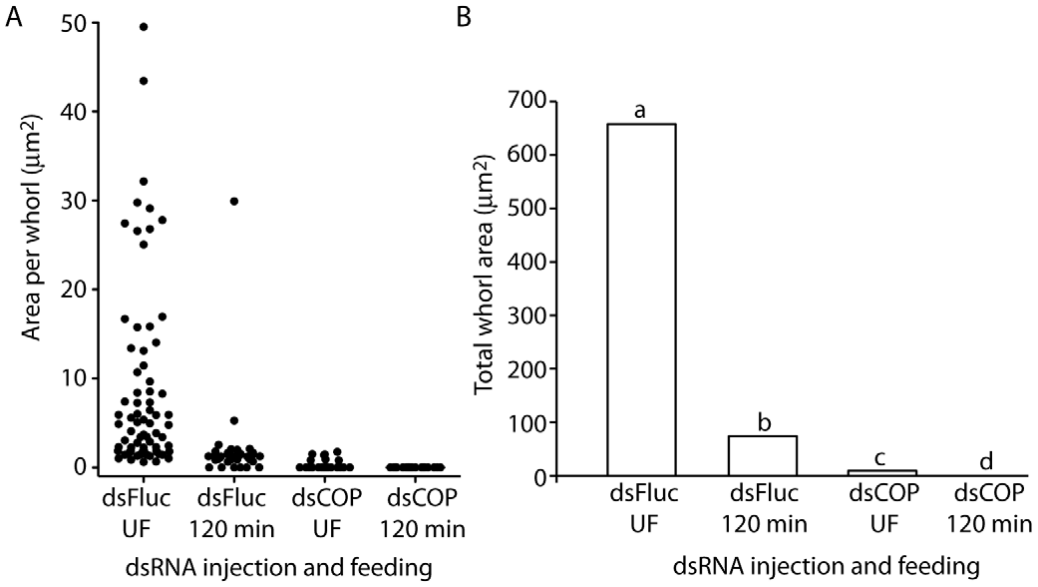
Quantitation of whorl size and number in dsRNA injected unfed and amino acid fed mosquitoes. Whorls with a minimum of five stacked ER membranes were counted and the total area of each whorl was determined as described in figure 2 legend. A) Size and number of RER whorls in 20 random fields from unfed and amino acid fed mosquitoes after 120 min. that were injected with Fluc or alpha-COPI dsRNA. B) Total whorl in the same sample as shown in A. Statistical analysis using Pearson Chi-square test revealed that the total whorl area in alpha-COPI dsRNA injected unfed and amino acid fed mosquitoes was significantly less than the total whorl area in Fluc dsRNA injected unfed mosquitoes (bars with different letters signify significance at p<0.001). In addition, the total whorl area in Fluc dsRNA injected fed mosquitoes was found to be significantly less than in Fluc dsRNA injected unfed mosquitoes using the Pearson Chi-square test (bars with different letters signify significance at p<0.001).

As shown in Figure 6, EM analysis of representative midguts from alpha-COPI and Fluc dsRNA injected mosquitoes showed that while large characteristic RER whorls were present in the midguts of unfed Fluc dsRNA injected mosquitoes (Figures 6A, 6B), the RER present in unfed alpha-COPI dsRNA injected mosquitoes was disorganized and RER whorl structures were absent (Figures 6C, 6D). RER reorganization at 120 min post-feeding in Fluc dsRNA injected mosquitoes looked similar to that of uninjected mosquitoes at the same time point, in that few if any whorls were present (compare Figures 7B and 1D). As shown in Figure 8, by quantitating the number and size of whorls in midgut epithelial cells from alpha-COPI and Fluc dsRNA injected mosquitoes, it is clear that loss of alpha-COPI expression in unfed mosquitoes was associated with a significant decrease in RER whorl formation. Moreover, a similar quantitative analysis of midgut epithelial cells from amino acid fed alpha-COPI dsRNA injected mosquitoes, showed highly disorganized endosomal structures and extended regions of swollen RER (Figures 7C and 7D). Taken together, these data indicate that functional alpha-COPI protein is required for normal whorl formation in the midgut epithelial cells of unfed mosquitoes.

### Expression of late phase midgut proteases is inhibited in alpha-COPI deficient mosquitoes

**Figure 9:**
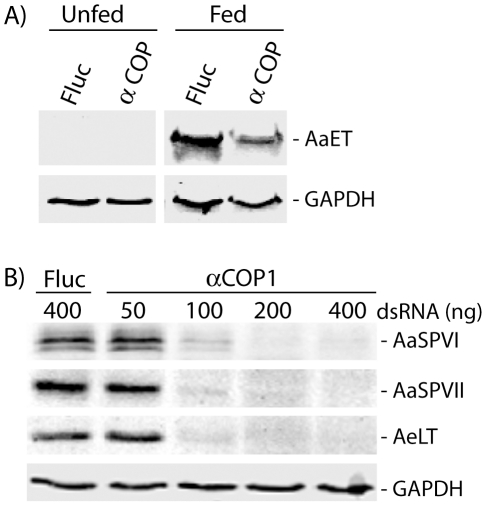
A deficiency in alpha-COPI inhibits feeding-induced expression of late phase midgut proteases. A) Representative Western blot of AeET protein expression in the midguts of mosquitoes injected with 400 ng of Fluc or alpha-COPI dsRNA and maintained on sugar for 3 days and then dissected (unfed), or blood fed after 3 days of sugar feeding and dissected at 3 hr PBM (fed). The GAPDH antibody was used as a protein loading control. Each lane contains the same midgut equivalents obtained from pooled mosquitoes. B) Representative Western blots of protein extracts prepared from pooled mosquito midguts dissected at 24 hr PBM and analyzed with antibodies against late phase serine proteases (AaSPVI, AaSPVII, AaLT) or GAPDH. Mosquitoes were injected with the indicated amount of dsRNA 3 days prior to blood feeding.

Blood meal digestion in *Ae. aegpti* mosquitoes requires the synthesis and secretion of numerous proteases, the best characterized of which are serine proteases. The early trypsin protease (AeET) is synthesized from pre-existing mRNA and secreted into the lumen within the first 6 hr PBM, whereas the late phase serine proteases AaSPVI, AaSPVII, and AaLT, are transcribed, translated, and secreted between 18–30 hr PBM [31]. To determine if alpha-COPI functions are required for the synthesis and secretion of early and late phase serine proteases, we injected mosquitoes with Fluc and alpha-COPI dsRNA 3 days prior to blood feeding and used Western blotting to detect protease protein expression at 3 hr PBM (AeET) and 24 hr PBM (AaSPVI, AaSPVII, AaLT). As shown in Figure 9A, the pattern of AeET protein expression is similar in Fluc and alpha-COPI dsRNA injected mosquitoes, suggesting that alpha-COPI functions are not required for early phase blood meal digestion. However, as shown in Figure 9B, 100–400 ng of alpha-COPI dsRNA blocks expression of all three abundant late phase proteases at 24 hr PBM in a dose-dependent manner. Lack of late phase serine protease expression in alpha-COPI deficient mosquitoes was associated with ejection of the undigested blood meal between 12–30 hr PBM (data not shown).

## Discussion

Blood feeding is required by *Ae. aegypti* mosquitoes to obtain the necessary protein-derived nutrients for completion of the gonotrophic cycle [32]. Understanding the biochemical and cellular regulation of blood meal metabolism will provide insights into this critical process in *Ae. aegypti*, as well as other blood-feeding arthropods that function as vectors of blood borne pathogens. Our objective in the studies reported here was to first determine if amino acids were sufficient to induce RER whorl unwinding, and if they were, to use a combination of proteomic and RNAi approaches to identify proteins that may be required for maintenance of RER whorls in midgut epithelial cells. It is possible that RER whorls function by providing a mechanism to rapidly activate midgut secretion pathways upon feeding, rather than expend energy maintaining them in the absence of feeding.

Biochemical studies have shown that in addition to globin, albumin, and immunoglobulin proteins, which make up 80% of the soluble proteins in human blood, free amino acids are also present in blood [21]. Based on physiological studies showing that artificial amino acids meals are sufficient to induce early events in the blood digestion process [33], it has been proposed that amino acids could play a signaling role by inducing protease expression [24]. Consistent with this idea, we have recently shown that amino acid feeding stimulates translation of pre-existing early trypsin mRNA through activation of the TOR signaling pathway [23]. Since large ER whorl structures have been shown to exist in the midgut of *Ae. aegypti* and other mosquito species [2], [3], [4], [5], [6], [8], [9], we used EM ultrastructural analysis to test if amino acid feeding induces ER whorl unwinding. As shown in Figures 1 and 2, our data clearly demonstrate that amino acid feeding does induce ER whorl unwinding, and moreover, that the most abundant whorls consist of rough ER membranes based on the high density of ribosomal particles.

Recently, several studies have reported the presence of ER whorl-like structures in cultured cells following different treatment conditions. For example, treatment of mouse Leydig cells with the piperazine derivative diethylcarbamazine citrate (DEC), led to the appearance of large lipid droplets, degenerative mitochondria, and giant smooth ER whorls in some cells [25]. Since DEC has been shown to disrupt ER and golgi vesicle transport processes, it could explain the formation of ER whorls. A similar loss of function study leading to the formation of ER whorls was seen in human HeLa cells in which expression of the ER protein Yip1A was knocked down by RNAi [18]. Therefore one of the functions of Yip1A could be to maintain proper ER/golgi membrane networks under normal conditions, but when it is absent, ER membranes collapse into whorl structures.

Another explanation for whorl formation is the presence of one or more abundant ER-associated proteins that stabilize stacked ER membranes in the absence of ongoing protein synthesis. Support for this model comes from studies in cultured cells showing that protein overexpression can induce ER whorl formation. Snapp et al. (2003) [15] reported that overexpression of cytochrome b5 induced the formation of smooth ER whorls, they called organized smooth ER (OSER), in transfected CV-1 cells. These same OSER structures could be observed when b5-GFP fusion proteins were over expressed, as long as they retained dimerization function in either the b5 or GFP protein domains. The authors proposed that weak noncovalent interactions between ER resident proteins on apposing stacked membranes are sufficient to maintain smooth ER whorl structures. Similar OSER structures were shown to form in HeLa cells that were infected with an adenovirus vector expressing the ER resident protein LAT linked to a heterologous protein dimerization domain [16], or overexpression of a viral protein that associates with ER membranes in infected cells [17].

In order to identify candidate RER whorl forming proteins in *Ae aegypti* midgut epithelial cells, we took advantage of the fact that an amino acid meal is sufficient to induce whorl unwinding, which simplifies both EM analysis and protein identification by mass spectrometry because it eliminates interference from abundant blood meal proteins. Moreover, since we found that RER whorl unwinding occurs rapidly once feeding begins (Figure 1), we were able to use a short recovery time of only 30 min to enrich for microsomal proteins that are present in the mosquito midgut prior to feeding. The LC-MS/MS analysis identified 127 proteins using microsomal midgut protein samples from unfed and amino acid fed mosquitoes. Eight proteins were considered candidate whorl associated proteins based on their known location and function in ER and Golgi membrane compartments (see Table 1). Since three of the candidate proteins encoded COPI coatomer subunits, and the peptide frequency was lower for each of the proteins in samples from fed mosquitoes compared to unfed mosquitoes, we chose alpha-COPI as a representative coatomer subunit and performed RNAi knockdown experiments. Data presented in Figures 6, 7, and 8 show that loss of alpha-COPI expression is associated with the absence of RER whorls in unfed mosquitoes, as well as membrane disorganization and ER swelling in midgut epithelial cells of amino acid fed mosquitoes. These data suggest that alpha-COPI, and likely two or more of the other seven COPI coatomer subunits, are involved in RER whorl formation and maintenance in midgut epithelial cells of unfed female mosquitoes. Based on the association of RER whorls with untranslated AeET mRNA transcripts in unfed mosquitoes, we predicted that RER whorls inhibited the translation of AaET transcripts. However the data in Figure 9A clearly show that this is not the case since sugar fed alpha-COPI dsRNA injected mosquitoes lacked both RER whorls and AaET protein expression. Surprisingly, while synthesis and secretion of AeET was not altered in blood fed alpha-COPI deficient mosquitoes, expression of three abundant late phase proteases (AaSPVI, AaSPVII, AaLT) was inhibited (Figure 9B), indicating that COPI vesicle transport is required for later events in the blood digestion process.

The COPI vesicle transport system has been shown to function in most cells as an retrograde transport mechanism that returns golgi-modified proteins back to the ER where they function in cell signaling and metabolism [29], [30]. However, the COPI system has also been shown to function in anterograde transport in some secretory cells where it was found to be required for exocytosis of specific proteins [34]. The COPI vesicle transport system consists of seven coatomer subunits (alpha, beta, beta', gamma, delta, epsilon, zeta), which function as structural components that promote vesicle formation, a G protein that facilitates coatomer assembly and membrane budding (Arf), guanine nucleotide exchange factors (GEFs) that activate Arf proteins and thereby initiate coatomer assembly, and GTPase activating proteins (ArfGAPs) that stimulate GTP hydrolysis in Arf proteins and induce coatomer disassembly, which is required for vesicle membrane fusion. Based on our finding that loss of alpha-COPI expression in midgut epithelial cells of unfed mosquitoes disrupts RER whorl formation, without decreasing the total amount of RER membrane in cells (Figure 6C and 6D), we propose that COPI coatomer proteins directly contribute to whorl formation through subunit assembly. Further experiments are needed to directly test this idea, for example, by using immunogold EM analysis to determine if COPI coatomer proteins are tightly associated with RER whorls, and if so, which coatomer subunits are colocalized.

## Materials and Methods

### Mosquitoes

*Aedes aegypti* (L.) (Rockefeller strain) mosquitoes were used for all studies. Larvae were maintained on a diet consisting of equal proportions of rat chow (Sunburst Pet Foods, Phoenix, AZ), lactalbumin hydrolysate (USB, Cleveland, OH), and yeast hydrolysate (USB, Cleveland, OH). Female pupae were separated from males using a mosquito separator. Adult mosquitoes were routinely maintained at 28°C, 70–80% relative humidity and a photoperiod of 16∶8 h (L∶D), on 10% sucrose *ad libitum*.

### Preparation of amino acids meal or feeding buffer for mosquito feeding

Amino acid meals was prepared according to Noriega et al. [33] with modifications. Briefly, the amino acids meal consisted of 40 ml of amino acid-deficient M199 media (dM199) (Invitrogen Corporation, Carlsbad, CA), 1.6 ml of 100× MEM nonessential amino acid solution (Mediatech, Inc., Herndon, VA), 3.2 ml of 50× MEM amino acid solution (Mediatech, Inc.), and 10 mg of HEPES (Sigma, St. Louis, MO) (pH 7.2), with a final concentration of 2.3 mg/ml total amino. Just before feeding mosquitoes, ATP (Sigma) was added to the meal to a final concentration of 5 mM. The feeding buffer was composed of 100 mM NaHCO_3_ and 150 mM NaCl, pH 7.2, as described by Kogan [35].

### Preparation of midguts for transmission electron microscopy

Dissected midgets were fixed in 2.5% glutaraldehyde+2% formaldehyde in 0.1 M PIPES buffer (pH 7.4) for 1 hr at room temperature, washed in buffer, post-fixed in 1% osmium tetroxide in buffer for 1 hr, washed in deionized water, and stained with 2% aqueous uranyl acetate for 30 mins. Specimens were dehydrated through an ethyl alcohol series, infiltrated with Spurr's resin and flat embedded at 60°C. Longitudinal sections (50 nm) were cut on a Leica UC2T ultramicrotome onto uncoated 150 mesh copper grids, counter-stained with lead citrate, and viewed in an FEI CM12S electron microscope operated at 80 kV. TIFF mages (8 bit) were collected via an AMT 4 M pixel camera and used for image analysis.

### Whorl quantitation and statistical analysis

Twenty sequential and adjacent visual fields from each EM slide were imaged at a magnification of 8800. Total area of the ER whorls in 20 visual fields of electron microscope was measured with ImageJ (NIH), which covered a total area of 3335 µm^2^. Only ER membrane structures containing more than four membrane stacks were considered to be whorls and used for area measurements. The data were statistically analyzed as pixels per 20 visual fields using Pearson Chi-square test with SPSS for Windows (v11.5).

### Western Blots

Western blots of early phase (AeET) and late phase (AaSPVI, AaSPVII, AaLT) protease expression in unfed and blood fed dsRNA injected mosquitoes were performed as previously described [31]. Analysis of KDEL containing proteins by Western blotting was done by dissecting fifty midguts from amino acid fed or unfed (sugar fed) female mosquitoes in pre-cold PBS buffer, dipped into 7×Protease Inhibitor Cocktail in 100 mM phosphate buffer (pH 7.0) (Roche Applied Science, Germany) with a forceps, and transferred into an ice-cold 1.5-ml Eppendorf tube containing 60 µl of PBS/TDS buffer (1% Triton X-100, 12 mM Na deoxycholate, 0.2% SDS in PBS, and Protease Inhibitor Cocktail). The dissected midguts were homogenized using a blue Kontes pestle. The homogenate was incubated on ice for 10 min and then spun at 10000 rpm for 10 min at 4°C. The supernatant was transferred to a pre-chilled 1.5-ml tube and 5×SDS sample loading buffer was added. The mixture was boiled for 4 min, chilled on ice for 2 min, and then spun at 13000 rpm for 5 min at room temperature. The yielded supernatant was stored at −20°C for SDS-PAGE.

Protein samples normalized to equal midgut equivalents were separated on 12% SDS-PAGE using standard procedures. PageRuler™ Prestained Protein Ladder (Fermentas, USA) was used as a protein standard. The proteins were transferred onto Odyssey Nitrocellulose Membranes (LI-COR Inc. Lincoln, Nebraska, USA). The membranes were dried in the air for 1 h and blocked at room temperature with 4% nonfat dry milk in 25 mM Tris-HCl, pH 7.6, 150 mM NaCl, and 10% Tween-20 (TBST), and then incubated with mouse monoclonal antibody against the peptide sequence SEKDEL conjugated to KLH (Abcam Inc. Cambridge, MA, USA). Loading controls were performed using rabbit polyclonal antibody against full length native GAPDH protein from human erythrocytes (Abcam, USA) at 1∶1000 dilution in 4% nonfat milk TBST solution at 4°C overnight. After washing with TBS containing 0.1% Tween-20 (TBST), the membranes were incubated with goat anti-rabbit IRDye® 800CW or goat anti-mouse IRDye® 800CW secondary antibody (LI-COR Biosciences, USA) at a dilution of 1∶10000 in TBST/4% nonfat dry milk for 1 h at room temperature. Labeled proteins were visualized using Odyssey Infrared Imaging System (LI-COR Biosciences, USA).

### Microsomal midgut protein extraction

About 40 midguts from the fed or unfed female mosquitoes were dissected with pre-cold PBS buffer, dipped into 7×Protease Inhibitor Cocktail in 100 mM phosphate buffer (pH 7.0) (Roche Applied Science, Germany) with a forceps, and transferred into an ice-cold 1.5-ml Eppendorf tube containing 100 µl of 1×Isotonic Extraction Buffer (5 mM HEPES;pH 7.8, 0.25 M sucrose, 1 mM EGTA, 25 mM KCl), and an appropriate amount of Protease Inhibitor Cocktail. Mosquito midgut ER protein extraction was performed according using the Endoplasmic Reticulum Isolation kit (ER0100; Sigma, St. Louis, USA) with modification. Briefly, the dissected midguts were homogenized with a blue Kontes pestle and spun at 1000g for 10 min at 4°C. The post nuclear supernatant was transferred into a clean 1.5-ml tube and spun at 12000g for 15 min at 4°C. The post mitochondrial supernatant was transferred into a clean 1.5-ml tube and mixed with 7.5 volumes of 8 mM CaCl_2_ by vortexing. The mixture was incubated on ice for 10 min and then spun at 8,000*g* for 10 minutes at 4°C. The supernatant was discarded, and the pellet containing the microsomal fraction, was resuspended in 20 µl of 1× Isotonic Extraction Buffer followed by a 10-min of incubation on ice. A 5 µl portion of the extracted ER protein fraction was used for measuring protein concentration with the BCA™ Protein Assay Kit (Pierce, USA). The remaining ER proteins were mixed with an appropriate amount of 5×SDS sample loading buffer, boiled for 4 min, and spun at 13000× rpm for 5 min. The supernatant was stored at −20C for SDS-PAGE.

### SDS-PAGE of ER proteins and in-gel digestion

Equal amounts of ER proteins isolated from midguts of unfed and fed mosquitoes were separated by 12% SDS-PAGE and visualized using the GelCode® Blue Staining Kit (Thermo Scientific, Rockford, IL, USA). Each gel lane was cut into 4 equal slices using a scalpel, and labeled as A, B, C, and D from high to low molecular weight regions. The gel slices were stored individually stored in a clean Eppendorf tube for in-gel digestion. Each gel slice was washed first with ddH_2_O for 15 min, then twice with 50% acetonitrile (ACN), and finally with 100 mM ammonium bicarbonate (Ambic, pH 8.0) containing 50% ACN for 15 min. After drying, the gel pieces were subjected to the standard in-gel digestion protocol. Briefly, proteins were reduced by 10 mM DTT/100 mM Ambic at 56°C for 45 min., and alkylated by 55 mM iodoacetamide (IAA)/100 mM Ambic at room temperature in the dark for 30 min. Gel pieces were washed with 100 mM Ambic dehydrated with ACN and dried. Proteolytic digestion was performed with 12.5 ng/µl trypsin dissolved in 100 mM Ambic and incubated on ice for 45 min. The digested mixture was acidified with 2% trifluoroacetic acid (TFA) in water for 1–2 minutes and then the supernatant was collected in a clean 1.5-ml tube. The peptides were extracted from the gel slice using 0.1% TFA in water, 0.1% TFA in 30% ACN, and 0.1% TFA in 60% ACN, respectively, with ultrasonication. The peptides extracted in the four steps were combined together, concentrated by a SpeedVac to a desired volume and subjected to LC-MS/MS analysis.

### LC-MS/MS and data analysis

Mass spectrometry analysis was performed by the Chemistry & Biochemistry department proteomics core facility using in-house protocols. Briefly, trypsin digested protein samples were acidified with TFA and diluted to 20 µl prior to separation by C18 column (75 um×1 mm, LC Packings, Amsterdam, Netherlands) at a flow rate of 30–500 nl/min and introduced into Finnigan LTQ (Thermo electron corporation) through nano spray (2.8 kV). Liquid chromatography mobile phases consisted of Solution A (90% water, 10%methonal, 0.5% formic acid, 0.01% TFA) and Solution B (98% methanol, 2% water, 0.5% formic acid, 0.01% TFA). A 120-min linear gradient from 0 to 90% B was typically used. Samples were subjected to nanoelectrospray mass spectrometry using standard procedures and data were analyzed using Sequest (ThermoFinnigan, San Jose, CA; version27, rev. 12) and X! Tandem (www.thegpm.org; version 2007.01.01.1) using the *Aedes aegypti* database. Scaffold (Proteome Software Inc., Portland, OR) was used to validate MS/MS based peptide and protein identifications. Sequest identifications required at least ΔCn scores of greater than 0.08 and XCorr scores of greater than 1.8, 2.5, 3.5 for singly, doubly, triply charged peptides. X! Tandem identifications required at least −Log(Expect Scores) scores of greater than 3.0.

### Protein annotation analysis

ER proteins listed in Table 1 and Table S1were annotated according to the UniProtKB/Swiss-Prot (http://www.expasy.ch/sprot/sprot_details.html), European Bioinformatics Institute (EBI) (http://www.ebi.ac.uk/) or UniProtKB/TrEMBL (http://www.ebi.ac.uk/trembl/) knowledge databases. Protein entries from the midgut ER were BLASTp-searched http://www.ncbi.nlm.nih.gov/BLAST/ in the Swiss-Prot database http://www.expasy.ch. Homologue protein entries (E-values<0.01) were subjected to ontology analysis using the PANDORA server http://www.pandora.cs.huji.ac.il/.

### RNAi-mediated knockdown of alpha-COPI expression and validation by QRTPCR

alpha-COPI gene was subjected to a RNAi according to our previous study [31]. Briefly, a DNA fragment of alpha-COPI gene was amplified by PCR (forward primer containing the T7 promoter sequence: 5′ TAATACGACTCACTATAGGGAGATGCTGACAAATTTCGAAACCAA 3′; and reverse primers containing the T7 promoter sequence: 5′ TAATACGACTCACTATAGGGAGATCCGTCGCCGTAGGATTCTT 3′) and cloned into the pGEM-T easy vector (Promega). Subsequently, a double-strand RNA (dsRNA) was synthesized *in vitro* transcription using the MEGAscript RNAi Kit (Ambion). Two-day old female mosquitoes were injected with 400 ng of dsRNA using a Nanoject II microinjector (Drummond Scientific). The knockdown efficiency of mRNA encoding alpha-COPI was determined using mosquitoes injected with Fluc (firefly luciferase) dsRNA as a control. QRT-PCR was performed using PerfeCTa SYBR Green FastMix (Quanta BioSciences) by Real-Time PCR (7300 Real-Time PCR System, Applied Biosystems) with alpha-COPI forward primer: 5′ GTGTCCGCATCGTTGGATCA 3′, alpha-COPI reverse primer: 5′ ACAACAGCATCAGCTTGCCCAA 3′. Ribosomal protein S7 mRNA levels were used as an internal control for normalization. Statistical analysis of the gene expression was done by unpaired student *t* test using GraphPad Prism software (GraphPad Software, Inc.).

## Supporting Information

Table S1 Proteomic analysis of *Ae. aegypti* midgut microsomal proteins isolated from unfed (sugar fed) and amino acid fed (30 min. post-feeding).
(DOC)
