# Supplementary material for: Alpha-COPI Coatomer Protein Is Required for Rough Endoplasmic Reticulum Whorl Formation in Mosquito Midgut Epithelial Cells
Source: PLoS One. 2011 Mar 31;6(3):e18150. doi: 10.1371/journal.pone.0018150 (PMC3069061; doi:10.1371/journal.pone.0018150)
Supplement: Table S1 — Proteomic analysis of Ae. aegypti midgut microsomal proteins isolated from unfed (sugar fed) and amino acid fed (30 min. post-feeding). (DOC) [file pone.0018150.s001.doc]

Supplemental Table 1. Proteomic analysis of midgut microsomal proteins in unfed and amino acid fed (30 min.) *Aedes aegypti* mosquitoes.

| **Identified Proteins** | **Accession Number** | **Mol. Wt.** | **Unfed** | **Fed** | **Cellular Component** | **Biological Process** | **Mol. Function** |
| --- | --- | --- | --- | --- | --- | --- | --- |
| fimbrin/plastin | gi|157167757 | 78 kDa | 8 | 4 | Actin cytoskeleton | Response to osmotic stress/Actin filament organization | Actin binding/Calcium ion binding |
| fructose-bisphosphate aldolase | gi|157111186 | 39 kDa | 2 | 0 | Actin cytoskeleton | Glycolysis | Aldolase activity/ATPase binding |
| vinculin | gi|157111402 | 107 kDa | 0 | 0 | Actin cytoskeleton | Cell adhesion | Structural molecule activity/Actin binding |
| phosphofructokinase | gi|157114499 | 87 kDa | 3 | 4 | Apical plasma membrane | Glycolysis | ATP binding |
| lethal giant larva, putative | gi|157124574 | 129 kDa | 0 | 3 | Cell junction | Larval development | Binding |
| clathrin heavy chain | gi|157136693 | 192 kDa | 0 | 0 | Clathrin-coat of coated pit/of trans-Golgi network vesicle | Vesicle-mediated transport/Introcellular protein transport | Structural molecule activity/Protein binding |
| low-density lipoprotein receptor (ldl) | gi|157127000 | 227 kDa | 0 | 0 | Clathrin-coated endocytic vesicle membrane | Endocytosis/Cholesterol homeostasis | Calcium ion binding/Receptor activity |
| past-1 | gi|157126105 | 61 kDa | 3 | 0 | Coated pit, endocytic vesicle | Vesicle organization and biogenesis | Calcium ion binding/GTP binding |
| activated protein kinase C receptor | gi|94468452 | 35 kDa | 5 | 6 | COPI vesicle coat | Golgi vesicle transport/Protein secretion | Receptor activity/Kinase activity |
| coatomer beta subunit | gi|157106589 | 107 kDa | 8 | 4 | COPI vesicle coat | Intracellular protein transport/Vesicel-mediated transport | Structural molecule activity/Protein binding |
| coatomer | gi|157112032 | 106 kDa | 6 | 0 | COPI vesicle coat | ER to Golgi vesicle-mediated transport/Protein transport | Structural molecule activity/ATP binding |
| coatomer | gi|157134451 | 139 kDa | 7 | 0 | COPI vesicle coat | Vesicle-mediated transport/Introcellular protein transport | Structural molecule activity/Protein binding |
| eukaryotic translation elongation factor | gi|157106353 | 94 kDa | 21 | 15 | Cytoplasm | Translation elongation | GTP binding/GTPase activity |
| translation elongation factor EF-1 alpha/Tu | gi|94468780 | 50 kDa | 13 | 10 | Cytoplasm | Translation elongation | GTP binding/GTPase activity |
| heat shock cognate 70 | gi|94468966 | 71 kDa | 16 | 18 | Cytoplasm | Response to stress | ATP binding |
| heat shock protein . | gi|157130209 | 82 kDa | 12 | 10 | Cytoplasm | Response to stress | ATP/Protein binding |
| carbonic anhydrase | gi|157108024 | 31 kDa | 7 | 6 | Cytoplasm | One-carbon metabolic process | Zinc ion binding |
| eukaryotic translation initiation factor 3, theta subunit | gi|157115084 | 133 kDa | 11 | 9 | Cytoplasm | Formation of translation initiation complex | Structural molecule activity/ATP,protein binding |
| elongation factor 1-gamma | gi|94468844 | 49 kDa | 7 | 7 | Cytoplasm | Translation elongation | Protein binding |
| annexin | gi|94468944 | 36 kDa | 7 | 6 | Cytoplasm | Cell-cell adhesion | Calcium ion binding/Phospholipid binding |
| 14-3-3 protein sigma, gamma, zeta, beta/alpha | gi|157128417 | 29 kDa | 7 | 6 | Cytoplasm | DNA damage response/Signal transduction | Protein binding |
| elongation factor 1-beta2 | gi|157112332 | 22 kDa | 6 | 7 | Cytoplasm | Translation elongation | Translation elongation factor activity |
| translation initiation factor 3, subunit S9, putative | gi|157116712 | 80 kDa | 8 | 7 | Cytoplasm | Translation initiation | RNA, necleotide binding/Factor activity |
| proliferation-associated 2g4 (pa2g4/ebp1) | gi|157132023 | 48 kDa | 6 | 7 | Cytoplasm | Cell proliferation/rRNA processing | RNA binding/Transcription factor activity |
| translation initiation factor 3, subunit S8, putative | gi|157117704 | 106 kDa | 6 | 4 | Cytoplasm | Translation initiation | Translation initiation factor activity |
| multifunctional 14-3-3 family chaperone | gi|94468884 | 28 kDa | 4 | 5 | Cytoplasm | Response to stress | Binding |
| heterogeneous nuclear ribonucleoprotein | gi|157110306 | 32 kDa | 3 | 5 | Cytoplasm | Nuclear mRNA splicing | Nucleic acid binding |
| dendritic cell protein | gi|157105605 | 44 kDa | 5 | 4 | Cytoplasm | Endocytosis/Immune response | Calcium ion binding/Protein binding |
| chaperonin-60kD, ch60 | gi|157129785 | 61 kDa | 7 | 0 | Cytoplasm | Protein folding | Protein binding/ATP binding |
| adenylyl cyclase-associated protein | gi|157168009 | 73 kDa | 5 | 0 | Cytoplasm | Cytoskeleton organization | Actin binding |
| elongation factor 1 beta/delta chain | gi|94468960 | 29 kDa | 0 | 3 | Cytoplasm | Translation elongation | Translation elongation factor activity |
| eukaryotic translation initiation factor 3 subunit | gi|157134639 | 36 kDa | 4 | 3 | Cytoplasm | Translation | RNA binding |
| eukaryotic translation initiation factor | gi|157136899 | 41 kDa | 3 | 4 | Cytoplasm | Translation | Translation initiation factor activity |
| gmp synthase | gi|157124722 | 76 kDa | 5 | 3 | Cytoplasm | Glutamine metabolic process/GMP biosynthetic process | ATP binding/GMP synthase activity |
| 67 kDa polymerase-associated factor PAF67, putative | gi|157123722 | 63 kDa | 4 | 0 | Cytoplasm | Translation initiation | Protein binding |
| translation initiation factor 3 subunit h | gi|94468864 | 35 kDa | 0 | 0 | Cytoplasm | Translation | Translation initiation factor activity |
| translation initiation factor 3 subunit e | gi|94468984 | 51 kDa | 0 | 6 | Cytoplasm | Translation | Translation initiation factor activity |
| lysyl-tRNA synthetase | gi|157106533 | 66 kDa | 4 | 3 | Cytoplasm | Lysyl-tRNA aminoacylation | ATP binding/Ligase activity |
| chaperonin | gi|157116575 | 53 kDa | 3 | 0 | Cytoplasm | Protein folding | ATP binding/Protein binding |
| arginyl-tRNA synthetase | gi|157117728 | 76 kDa | 3 | 3 | Cytoplasm | Arginyl-tRNA aminoacylation | ATP binding/Ligase activity |
| eukaryotic translation initiation factor 3 subunit | gi|157124565 | 68 kDa | 4 | 0 | Cytoplasm | Translation initiation | Translation initiation factor activity |
| hypothetical protein AaeL_AAEL000328 | gi|157130058 | 44 kDa | 0 | 0 | Cytoplasm | Oxidation reduction | Zinc ion binding |
| myosin vi | gi|157105157 | 143 kDa | 0 | 0 | Cytoplasm | Actin filament-based movement | Actin binding/ATP binding/Motor activity |
| ATP-citrate synthase | gi|157105391 | 123 kDa | 3 | 0 | Cytoplasm | Metabolic process | ATP binding/Ligase activity |
| eukaryotic translation initiation factor 3 subunit 5 epsilon-like protein | gi|94468862 | 32 kDa | 0 | 0 | Cytoplasm | Translation | Factor activity |
| glutaminyl-trna synthetase | gi|157167670 | 88 kDa | 0 | 3 | Cytoplasm | Aminoacylation | ATP binding/Ligase activity |
| elongation initiation factor 5C | gi|94468852 | 48 kDa | 0 | 0 | Cytoplasm | Translation elongation | Factor activity |
| leucyl-tRNA synthetase | gi|157113359 | 135 kDa | 2 | 0 | Cytoplasm | Aminoacylation | ATP binding/Ligase activity |
| methionine aminopeptidase | gi|157107323 | 56 kDa | 0 | 0 | Cytoplasm | Proteolysis/Cellular process | Cobalt ion binding/Aminopeptidase activity |
| purine biosynthesis protein 6, pur6 | gi|157137371 | 47 kDa | 0 | 0 | Cytoplasm | De novo IMP biosynthetic process | ATP binding |
| chaperonin | gi|157116665 | 57 kDa | 0 | 0 | Cytoplasm | Protein folding | ATP binding/Unfolded protein binding |
| carboxylase:pyruvate/acetyl-coa/propionyl-coa | gi|157123846 | 131 kDa | 0 | 0 | Cytoplasm | Gluconeogenesis | ATP binding/Biotin binding |
| actin | gi|677901 | 42 kDa | 8 | 7 | Cytoskeleton | Cell motion | Structural constituent of cytoskeleton/ATP,Protein binding |
| ATP synthase alpha subunit vacuolar | gi|157119815 | 68 kDa | 17 | 9 | Cytosol | ATP synthesis coupled proton transport | ATP binding |
| initiation factor EIF-4A | gi|94468792 | 46 kDa | 5 | 5 | Cytosol | Translation | ATP, RNA binding |
| polyadenylate-binding protein | gi|94468824 | 70 kDa | 0 | 2 | Cytosol | RNA splicing | RNA binding/Nucleotide binding |
| casein kinase ii, alpha chain (cmgc group iv) | gi|157131473 | 41 kDa | 0 | 0 | Cytosol | Protein AA phosphorylation | ATP binding/Kinase activity |
| utp-glucose-1-phosphate uridylyltransferase 2 | gi|157110519 | 58 kDa | 0 | 0 | Cytosol | Metabolic process | Nucleotidyltransferase activity/Protein binding |
| nadp-specific isocitrate dehydrogenase | gi|157133019 | 37 kDa | 0 | 0 | Cytosol | Metabolic process/Oxidation reduction | Dehydrogenase activity/Mg2+ binding/NAD/NADH binding |
| signal recognition particle 68 kda protein . | gi|157125066 | 70 kDa | 2 | 0 | Endoplasmic reticulum | Response to drug | RNA binding/Signal recognition |
| ER protein disulfide isomerase | gi|94468800 | 56 kDa | 3 | 0 | Endoplasmic reticulum | Cell redox homeostasis | Isomerase activity |
| hypothetical protein AaeL_AAEL009955 | gi|157124742 | 367 kDa | 4 | 2 | Extracellular region | Lipid transport | Fatty acid binding/lipid transporter activity |
| alpha-amylase | gi|157132559 | 60 kDa | 0 | 0 | Extracellular region | Carbohydrate metabolic process | Cation binding/catalytic activity |
| ebna2 binding protein P100 | gi|157127091 | 103 kDa | 22 | 26 | Golgi apparatus | Transcription | Transcription cofactor/NA or protein binding |
| hypothetical protein AaeL_AAEL002178 | gi|157167507 | 48 kDa | 0 | 0 | Golgi apparatus | Metabolic process | ATP binding/Ligase activity |
| na+/k+ atpase alpha subunit | gi|157131373 | 111 kDa | 4 | 4 | Integral to membrane | ATP biosynthetic process | ATP binding/ATPase activity |
| abc transporter, putative | gi|157125068 | 69 kDa | 0 | 0 | Integral to membrane | Heavy metal ion transport | ATP binding/Iron-sulfur cluster binding/iron ion binding |
| tubulin beta chain | gi|157132376 | 50 kDa | 17 | 15 | Microtubule | Microtubule-based movement | GTP binding/GTPase activity |
| alpha tubulin | gi|94468850 | 50 kDa | 10 | 9 | Microtubule | Microtubule-based movement | GTP binding/GTPase activity |
| tubulin beta chain | gi|157108658 | 51 kDa | 3 | 0 | Microtubule | Microtubule-based movement/Protein polymerization | Structural molecule activity/GTP binding |
| 2-oxoglutarate dehydrogenase | gi|157114121 | 119 kDa | 19 | 12 | Mitochondrion | Glycolysis | Thiamin pyrophosphate binding/Protein binding |
| glutamate dehydrogenase | gi|157126236 | 61 kDa | 14 | 11 | Mitochondrion | Cellular AA metabolic process | ADP, ATP, GTP, NAD binding |
| 2-oxoglutarate dehydrogenase | gi|157109931 | 102 kDa | 12 | 4 | Mitochondrion | Glycolysis | Thiamin pyrophosphate binding/Protein binding |
| saccharopine dehydrogenase | gi|157106746 | 103 kDa | 9 | 4 | Mitochondrion | Metabolic process/Oxidation reduction | Binding/Electron carrier activity |
| ATP synthase subunit beta vacuolar | gi|157111261 | 55 kDa | 9 | 4 | Mitochondrion | ATP synthesis coupled proton transport | ATP binding |
| dihydrolipoamide succinyltransferase component of 2-oxoglutarate dehydrogenase | gi|157131797 | 52 kDa | 4 | 4 | Mitochondrion | Tricarboxylic acid cycle | Lipoic acid binding/Protein binding |
| succinyl-coa synthetase beta chain | gi|157130278 | 49 kDa | 0 | 5 | Mitochondrion | Metabolic process/Oxidation reduction | ATP binding/Ligase activity |
| acyl-coa dehydrogenase | gi|157136755 | 47 kDa | 0 | 6 | Mitochondrion | Oxidation reduction | FAD binding/electron carrier activity |
| methionine-tRNA synthetase | gi|157106644 | 108 kDa | 3 | 5 | Mitochondrion | Methionyl-tRNA aminoacylation | ATP binding/Ligase activity |
| vacuolar ATP synthase subunit h | gi|157113604 | 55 kDa | 3 | 4 | Mitochondrion | ATP synthesis coupled proton transport | Hydrogen ion transmembrane transporter activity/ATP binding |
| vacuolar ATP synthase subunit E . | gi|94469084 | 26 kDa | 4 | 0 | Mitochondrion | ATP synthesis coupled proton transport | Protein binding/ATPase activity |
| F0F1-type ATP synthase beta subunit . | gi|94468834 | 54 kDa | 4 | 2 | Mitochondrion | ATP synthesis coupled proton transport | ATP binding/ATPase activity |
| mitochondrial aldehyde dehydrogenase . | gi|94469046 | 57 kDa | 5 | 0 | Mitochondrion | Oxidation reduction | Oxidoreductase activity |
| citrate synthase . | gi|157133341 | 52 kDa | 0 | 5 | Mitochondrion | Tricarboxylic acid cycle/metabolic process | Citrate synthase activity |
| dihydrolipoamide acetyltransferase component of pyruvate dehydrogenase . | gi|157105359 | 54 kDa | 0 | 0 | Mitochondrion | Glycolysis | Lipoic acid binding/Protein binding |
| spermatogenesis associated factor . | gi|157132226 | 80 kDa | 4 | 0 | Mitochondrion | Cell diferentiation/Spermatogenesis | ATP binding |
| 3-hydroxyacyl-coa dehyrogenase . | gi|157140862 | 79 kDa | 4 | 0 | Mitochondrion | Fatty acid beta-oxidation/Oxidation reduction | Coenzyme binding/Enzyme activity |
| mitochondrial ATP synthase alpha subunit . | gi|94468442 | 59 kDa | 3 | 0 | Mitochondrion | ATP synthesis coupled proton transport | Transporter activity/ATP binding |
| succinyl-CoA synthetase alpha subunit . | gi|94468890 | 34 kDa | 0 | 3 | Mitochondrion | Tricarboxylic acid cycle | Binding/Synthase activity |
| pyruvate dehydrogenase . | gi|157105561 | 39 kDa | 0 | 0 | Mitochondrion | Glycolysis/oxidation reduction | Pyruvate dehydrogenase activity |
| vacuolar H+-ATPase V1 sector subunit D . | gi|94469066 | 28 kDa | 0 | 0 | Mitochondrion | ATP synthesis coupled proton transport | ATPase activity/Protein binding |
| ferrochelatase . | gi|157109844 | 44 kDa | 0 | 0 | Mitochondrion | Heme biosynthetic process | Iron ion binding |
| nipsnap . | gi|157117700 | 32 kDa | 0 | 0 | Mitochondrion | Acetylation | Protein binding |
| isocitrate dehydrogenase . | gi|157134807 | 39 kDa | 0 | 0 | Mitochontrion | Oxidation reduction/Tricarboxylic acid cycle | NAD/NADH binding/Mg2+ binding |
| myosin i . | gi|157130852 | 119 kDa | 5 | 4 | Myosin complex-Cytoskeleton | mRNA transport/protein transport | ATP binding/motor activity |
| hypothetical protein AaeL_AAEL004472 . | gi|157106196 | 27 kDa | 0 | 0 | Nuclear preribosome | Ribosomal subunit assembly | RNA binding/ATP binding |
| nascent polypeptide associated complex alpha subunit (nac alpha) . | gi|157167407 | 17 kDa | 0 | 0 | Nucleus | Protein folding/transport | Protein binding |
| AMP dependent ligase . | gi|157111121 | 69 kDa | 4 | 0 | Nucleus | Metabolic process | ATP binding/Ligase activity/Protein binding |
| DEAD box ATP-dependent RNA helicase . | gi|157106194 | 68 kDa | 0 | 0 | Nucleus | Ribosomal subunit assembly | ATP binding/RNA binding |
| ribosomal protein L4 . | gi|94469276 | 49 kDa | 9 | 10 | Ribosome | Translation | Structural constituent of ribosome |
| 40S ribosomal protein SA . | gi|94468996 | 32 kDa | 9 | 8 | Ribosome | Translation | Structural constituent of ribosome |
| 40S ribosomal protein S3A . | gi|94468430 | 30 kDa | 3 | 6 | Ribosome | Translation | Structural constituent of ribosome |
| ribosomal protein L3 . | gi|94468802 | 47 kDa | 5 | 6 | Ribosome | Translation | Structural constituent of ribosome |
| 40S ribosomal protein S4 . | gi|94468404 | 30 kDa | 4 | 5 | Ribosome | Translation | Structural constituent of ribosome/RNA binding |
| 40S ribosomal protein S3 . | gi|157118131 | 28 kDa | 6 | 6 | Ribosome | Translation | Structural constituent of ribosome/RNA binding |
| 40S ribosomal protein S8 . | gi|94468438 | 23 kDa | 5 | 8 | Ribosome | Translation | Structural constituent of ribosome |
| ribosomal protein P0 . | gi|94468758 | 34 kDa | 5 | 6 | Ribosome | Translation elongation | Structural constituent of ribosome |
| ribosomal protein L7 . | gi|94468794 | 30 kDa | 3 | 5 | Ribosome | Translation | Structural constituent of ribosome |
| 60S ribosomal protein L18 . | gi|94468832 | 22 kDa | 3 | 0 | Ribosome | Translation | Structural constituent of ribosome |
| 60S ribosomal protein L5 . | gi|94469072 | 34 kDa | 4 | 3 | Ribosome | Translation | Structural constituent of ribosome/RNA binding |
| ribosomal protein S6 . | gi|157103333 | 37 kDa | 0 | 5 | Ribosome | Translation | Structural constituent of ribosome |
| ribosomal protein S4 . | gi|94468470 | 23 kDa | 5 | 5 | Ribosome | Translation | Structural constituent of ribosome/rRNA binding |
| ribosomal protein S5 . | gi|94468378 | 25 kDa | 2 | 5 | Ribosome | Translation | Structural constituent of ribosome/RNA binding |
| ribosomal protein L7AE . | gi|94468816 | 31 kDa | 2 | 3 | Ribosome | Ribosome biogenesis | Structural constituent of ribosome/RNA binding |
| 60S ribosomal protein L13a . | gi|94468830 | 27 kDa | 4 | 5 | Ribosome | Translation | Structural constituent of ribosome |
| 60S ribosomal protein L10A . | gi|94468570 | 25 kDa | 4 | 3 | Ribosome | Translation | Structural constituent of ribosome |
| ribosomal protein L15 . | gi|157133451 | 18 kDa | 3 | 3 | Ribosome | Translation | Structural constituent of ribosome |
| ribosomal protein L10 . | gi|94468384 | 25 kDa | 3 | 4 | Ribosome | Translation | Structural constituent of ribosome |
| ribosomal protein S2 . | gi|94468354 | 30 kDa | 0 | 4 | Ribosome | Translation | Structural constituent of ribosome/RNA binding |
| 60S ribosomal protein L2/L8 . | gi|94468892 | 29 kDa | 0 | 4 | Ribosome | Translation | Structural constituent of ribosome |
| cold induced protein (BnC24A), putative . | gi|157108695 | 16 kDa | 2 | 0 | Ribosome | Translation | Structural constituent of ribosome |
| ribosomal protein L9 . | gi|94468812 | 21 kDa | 3 | 0 | Ribosome | Translation | Structural constitient of ribosome/rRNA binding |
| 40S ribosomal protein S7 . | gi|157123489 | 22 kDa | 2 | 0 | Ribosome | Translation | Structural constitient of ribosome |
| 60S ribosomal protein L17 . | gi|94468826 | 22 kDa | 0 | 0 | Ribosome | Translation | Structural constituent of ribosome |
| 60S ribosomal protein L14 . | gi|94468474 | 21 kDa | 3 | 0 | Ribosome | Translation | Structural constituent of ribosome |
| ribosomal protein L19 . | gi|94468808 | 24 kDa | 0 | 0 | Ribosome | Translation | Structural constituent of ribosome |
| Identified Proteins (127) | Accession Number | Molecular Weight | Unfed | Fed | Cellular Component | Biological Process | Mol. Function |
| fimbrin/plastin . | gi|157167757 | 78 kDa | 8 | 4 | Actin cytoskeleton | Response to osmotic stress/Actin filament organization | Actin binding/Calcium ion binding |
| fructose-bisphosphate aldolase . | gi|157111186 | 39 kDa | 2 | 0 | Actin cytoskeleton | Glycolysis | Aldolase activity/ATPase binding |
| vinculin . | gi|157111402 | 107 kDa | 0 | 0 | Actin cytoskeleton | Cell adhesion | Structural molecule activity/Actin binding |
| phosphofructokinase . | gi|157114499 | 87 kDa | 3 | 4 | Apical plasma membrane | Glycolysis | ATP binding |
| lethal giant larva, putative . | gi|157124574 | 129 kDa | 0 | 3 | Cell junction | Larval development | Binding |
| clathrin heavy chain . | gi|157136693 | 192 kDa | 0 | 0 | Clathrin-coat of coated pit/of trans-Golgi network vesicle | Vesicle-mediated transport/Introcellular protein transport | Structural molecule activity/Protein binding |
| low-density lipoprotein receptor (ldl) . | gi|157127000 | 227 kDa | 0 | 0 | Clathrin-coated endocytic vesicle membrane | Endocytosis/Cholesterol homeostasis | Calcium ion binding/Receptor activity |
| past-1 . | gi|157126105 | 61 kDa | 3 | 0 | Coated pit, endocytic vesicle | Vesicle organization and biogenesis | Calcium ion binding/GTP binding |
| activated protein kinase C receptor . | gi|94468452 | 35 kDa | 5 | 6 | COPI vesicle coat | Golgi vesicle transport/Protein secretion | Receptor activity/Kinase activity |
| coatomer beta subunit . | gi|157106589 | 107 kDa | 8 | 4 | COPI vesicle coat | Intracellular protein transport/Vesicel-mediated transport | Structural molecule activity/Protein binding |
| coatomer . | gi|157112032 | 106 kDa | 6 | 0 | COPI vesicle coat | ER to Golgi vesicle-mediated transport/Protein transport | Structural molecule activity/ATP binding |
| coatomer . | gi|157134451 | 139 kDa | 7 | 0 | COPI vesicle coat | Vesicle-mediated transport/Introcellular protein transport | Structural molecule activity/Protein binding |
| eukaryotic translation elongation factor . | gi|157106353 | 94 kDa | 21 | 15 | Cytoplasm | Translation elongation | GTP binding/GTPase activity |
| translation elongation factor EF-1 alpha/Tu . | gi|94468780 | 50 kDa | 13 | 10 | Cytoplasm | Translation elongation | GTP binding/GTPase activity |
| heat shock cognate 70 . | gi|94468966 | 71 kDa | 16 | 18 | Cytoplasm | Response to stress | ATP binding |
| heat shock protein . | gi|157130209 | 82 kDa | 12 | 10 | Cytoplasm | Response to stress | ATP/Protein binding |
| carbonic anhydrase . | gi|157108024 | 31 kDa | 7 | 6 | Cytoplasm | One-carbon metabolic process | Zinc ion binding |
| eukaryotic translation initiation factor 3, theta subunit . | gi|157115084 | 133 kDa | 11 | 9 | Cytoplasm | Formation of translation initiation complex | Structural molecule activity/ATP,protein binding |
| elongation factor 1-gamma . | gi|94468844 | 49 kDa | 7 | 7 | Cytoplasm | Translation elongation | Protein binding |
| annexin . | gi|94468944 | 36 kDa | 7 | 6 | Cytoplasm | Cell-cell adhesion | Calcium ion binding/Phospholipid binding |
| 14-3-3 protein sigma, gamma, zeta, beta/alpha . | gi|157128417 | 29 kDa | 7 | 6 | Cytoplasm | DNA damage response/Signal transduction | Protein binding |
| elongation factor 1-beta2 . | gi|157112332 | 22 kDa | 6 | 7 | Cytoplasm | Translation elongation | Translation elongation factor activity |
| translation initiation factor 3, subunit S9, putative . | gi|157116712 | 80 kDa | 8 | 7 | Cytoplasm | Translation initiation | RNA, necleotide binding/Factor activity |
| proliferation-associated 2g4 (pa2g4/ebp1) . | gi|157132023 | 48 kDa | 6 | 7 | Cytoplasm | Cell proliferation/rRNA processing | RNA binding/Transcription factor activity |
| translation initiation factor 3, subunit S8, putative . | gi|157117704 | 106 kDa | 6 | 4 | Cytoplasm | Translation initiation | Translation initiation factor activity |
| multifunctional 14-3-3 family chaperone . | gi|94468884 | 28 kDa | 4 | 5 | Cytoplasm | Response to stress | Binding |
| heterogeneous nuclear ribonucleoprotein . | gi|157110306 | 32 kDa | 3 | 5 | Cytoplasm | Nuclear mRNA splicing | Nucleic acid binding |
| dendritic cell protein . | gi|157105605 | 44 kDa | 5 | 4 | Cytoplasm | Endocytosis/Immune response | Calcium ion binding/Protein binding |
| chaperonin-60kD, ch60 . | gi|157129785 | 61 kDa | 7 | 0 | Cytoplasm | Protein folding | Protein binding/ATP binding |
| adenylyl cyclase-associated protein . | gi|157168009 | 73 kDa | 5 | 0 | Cytoplasm | Cytoskeleton organization | Actin binding |
| elongation factor 1 beta/delta chain . | gi|94468960 | 29 kDa | 0 | 3 | Cytoplasm | Translation elongation | Translation elongation factor activity |
| eukaryotic translation initiation factor 3 subunit . | gi|157134639 | 36 kDa | 4 | 3 | Cytoplasm | Translation | RNA binding |
| eukaryotic translation initiation factor . | gi|157136899 | 41 kDa | 3 | 4 | Cytoplasm | Translation | Translation initiation factor activity |
| gmp synthase . | gi|157124722 | 76 kDa | 5 | 3 | Cytoplasm | Glutamine metabolic process/GMP biosynthetic process | ATP binding/GMP synthase activity |
| 67 kDa polymerase-associated factor PAF67, putative . | gi|157123722 | 63 kDa | 4 | 0 | Cytoplasm | Translation initiation | Protein binding |
| translation initiation factor 3 subunit h . | gi|94468864 | 35 kDa | 0 | 0 | Cytoplasm | Translation | Translation initiation factor activity |
| translation initiation factor 3 subunit e . | gi|94468984 | 51 kDa | 0 | 6 | Cytoplasm | Translation | Translation initiation factor activity |
| lysyl-tRNA synthetase . | gi|157106533 | 66 kDa | 4 | 3 | Cytoplasm | Lysyl-tRNA aminoacylation | ATP binding/Ligase activity |
| chaperonin . | gi|157116575 | 53 kDa | 3 | 0 | Cytoplasm | Protein folding | ATP binding/Protein binding |
| arginyl-tRNA synthetase . | gi|157117728 | 76 kDa | 3 | 3 | Cytoplasm | Arginyl-tRNA aminoacylation | ATP binding/Ligase activity |
| eukaryotic translation initiation factor 3 subunit . | gi|157124565 | 68 kDa | 4 | 0 | Cytoplasm | Translation initiation | Translation initiation factor activity |
| hypothetical protein AaeL_AAEL000328 . | gi|157130058 | 44 kDa | 0 | 0 | Cytoplasm | Oxidation reduction | Zinc ion binding |
| myosin vi . | gi|157105157 | 143 kDa | 0 | 0 | Cytoplasm | Actin filament-based movement | Actin binding/ATP binding/Motor activity |
| ATP-citrate synthase . | gi|157105391 | 123 kDa | 3 | 0 | Cytoplasm | Metabolic process | ATP binding/Ligase activity |
| eukaryotic translation initiation factor 3 subunit 5 epsilon-like protein . | gi|94468862 | 32 kDa | 0 | 0 | Cytoplasm | Translation | Factor activity |
| glutaminyl-trna synthetase . | gi|157167670 | 88 kDa | 0 | 3 | Cytoplasm | Aminoacylation | ATP binding/Ligase activity |
| elongation initiation factor 5C . | gi|94468852 | 48 kDa | 0 | 0 | Cytoplasm | Translation elongation | Factor activity |
| leucyl-tRNA synthetase . | gi|157113359 | 135 kDa | 2 | 0 | Cytoplasm | Aminoacylation | ATP binding/Ligase activity |
| methionine aminopeptidase . | gi|157107323 | 56 kDa | 0 | 0 | Cytoplasm | Proteolysis/Cellular process | Cobalt ion binding/Aminopeptidase activity |
| purine biosynthesis protein 6, pur6 . | gi|157137371 | 47 kDa | 0 | 0 | Cytoplasm | De novo IMP biosynthetic process | ATP binding |
| chaperonin . | gi|157116665 | 57 kDa | 0 | 0 | Cytoplasm | Protein folding | ATP binding/Unfolded protein binding |
| carboxylase:pyruvate/acetyl-coa/propionyl-coa . | gi|157123846 | 131 kDa | 0 | 0 | Cytoplasm | Gluconeogenesis | ATP binding/Biotin binding |
| actin . | gi|677901 | 42 kDa | 8 | 7 | Cytoskeleton | Cell motion | Structural constituent of cytoskeleton/ATP,Protein binding |
| ATP synthase alpha subunit vacuolar . | gi|157119815 | 68 kDa | 17 | 9 | Cytosol | ATP synthesis coupled proton transport | ATP binding |
| initiation factor EIF-4A . | gi|94468792 | 46 kDa | 5 | 5 | Cytosol | Translation | ATP, RNA binding |
| polyadenylate-binding protein . | gi|94468824 | 70 kDa | 0 | 2 | Cytosol | RNA splicing | RNA binding/Nucleotide binding |
| casein kinase ii, alpha chain (cmgc group iv) . | gi|157131473 | 41 kDa | 0 | 0 | Cytosol | Protein AA phosphorylation | ATP binding/Kinase activity |
| utp-glucose-1-phosphate uridylyltransferase 2 . | gi|157110519 | 58 kDa | 0 | 0 | Cytosol | Metabolic process | Nucleotidyltransferase activity/Protein binding |
| nadp-specific isocitrate dehydrogenase . | gi|157133019 | 37 kDa | 0 | 0 | Cytosol | Metabolic process/Oxidation reduction | Dehydrogenase activity/Mg2+ binding/NAD/NADH binding |
| signal recognition particle 68 kda protein . | gi|157125066 | 70 kDa | 2 | 0 | Endoplasmic reticulum | Response to drug | RNA binding/Signal recognition |
| ER protein disulfide isomerase . | gi|94468800 | 56 kDa | 3 | 0 | Endoplasmic reticulum | Cell redox homeostasis | Isomerase activity |
| hypothetical protein AaeL_AAEL009955 . | gi|157124742 | 367 kDa | 4 | 2 | Extracellular region | Lipid transport | Fatty acid binding/lipid transporter activity |
| alpha-amylase . | gi|157132559 | 60 kDa | 0 | 0 | Extracellular region | Carbohydrate metabolic process | Cation binding/catalytic activity |
| ebna2 binding protein P100 . | gi|157127091 | 103 kDa | 22 | 26 | Golgi apparatus | Transcription | Transcription cofactor/NA or protein binding |
| hypothetical protein AaeL_AAEL002178 . | gi|157167507 | 48 kDa | 0 | 0 | Golgi apparatus | Metabolic process | ATP binding/Ligase activity |
| na+/k+ atpase alpha subunit . | gi|157131373 | 111 kDa | 4 | 4 | Integral to membrane | ATP biosynthetic process | ATP binding/ATPase activity |
| abc transporter, putative . | gi|157125068 | 69 kDa | 0 | 0 | Integral to membrane | Heavy metal ion transport | ATP binding/Iron-sulfur cluster binding/iron ion binding |
| tubulin beta chain . | gi|157132376 | 50 kDa | 17 | 15 | Microtubule | Microtubule-based movement | GTP binding/GTPase activity |
| alpha tubulin . | gi|94468850 | 50 kDa | 10 | 9 | Microtubule | Microtubule-based movement | GTP binding/GTPase activity |
| tubulin beta chain . | gi|157108658 | 51 kDa | 3 | 0 | Microtubule | Microtubule-based movement/Protein polymerization | Structural molecule activity/GTP binding |
| 2-oxoglutarate dehydrogenase . | gi|157114121 | 119 kDa | 19 | 12 | Mitochondrion | Glycolysis | Thiamin pyrophosphate binding/Protein binding |
| glutamate dehydrogenase . | gi|157126236 | 61 kDa | 14 | 11 | Mitochondrion | Cellular AA metabolic process | ADP, ATP, GTP, NAD binding |
| 2-oxoglutarate dehydrogenase . | gi|157109931 | 102 kDa | 12 | 4 | Mitochondrion | Glycolysis | Thiamin pyrophosphate binding/Protein binding |
| saccharopine dehydrogenase . | gi|157106746 | 103 kDa | 9 | 4 | Mitochondrion | Metabolic process/Oxidation reduction | Binding/Electron carrier activity |
| ATP synthase subunit beta vacuolar . | gi|157111261 | 55 kDa | 9 | 4 | Mitochondrion | ATP synthesis coupled proton transport | ATP binding |
| dihydrolipoamide succinyltransferase component of 2-oxoglutarate dehydrogenase . | gi|157131797 | 52 kDa | 4 | 4 | Mitochondrion | Tricarboxylic acid cycle | Lipoic acid binding/Protein binding |
| succinyl-coa synthetase beta chain . | gi|157130278 | 49 kDa | 0 | 5 | Mitochondrion | Metabolic process/Oxidation reduction | ATP binding/Ligase activity |
| acyl-coa dehydrogenase . | gi|157136755 | 47 kDa | 0 | 6 | Mitochondrion | Oxidation reduction | FAD binding/electron carrier activity |
| methionine-tRNA synthetase . | gi|157106644 | 108 kDa | 3 | 5 | Mitochondrion | Methionyl-tRNA aminoacylation | ATP binding/Ligase activity |
| vacuolar ATP synthase subunit h . | gi|157113604 | 55 kDa | 3 | 4 | Mitochondrion | ATP synthesis coupled proton transport | Hydrogen ion transmembrane transporter activity/ATP binding |
| vacuolar ATP synthase subunit E . | gi|94469084 | 26 kDa | 4 | 0 | Mitochondrion | ATP synthesis coupled proton transport | Protein binding/ATPase activity |
| F0F1-type ATP synthase beta subunit . | gi|94468834 | 54 kDa | 4 | 2 | Mitochondrion | ATP synthesis coupled proton transport | ATP binding/ATPase activity |
| mitochondrial aldehyde dehydrogenase . | gi|94469046 | 57 kDa | 5 | 0 | Mitochondrion | Oxidation reduction | Oxidoreductase activity |
| citrate synthase . | gi|157133341 | 52 kDa | 0 | 5 | Mitochondrion | Tricarboxylic acid cycle/metabolic process | Citrate synthase activity |
| dihydrolipoamide acetyltransferase component of pyruvate dehydrogenase . | gi|157105359 | 54 kDa | 0 | 0 | Mitochondrion | Glycolysis | Lipoic acid binding/Protein binding |
| spermatogenesis associated factor . | gi|157132226 | 80 kDa | 4 | 0 | Mitochondrion | Cell diferentiation/Spermatogenesis | ATP binding |
| 3-hydroxyacyl-coa dehyrogenase . | gi|157140862 | 79 kDa | 4 | 0 | Mitochondrion | Fatty acid beta-oxidation/Oxidation reduction | Coenzyme binding/Enzyme activity |
| mitochondrial ATP synthase alpha subunit . | gi|94468442 | 59 kDa | 3 | 0 | Mitochondrion | ATP synthesis coupled proton transport | Transporter activity/ATP binding |
| succinyl-CoA synthetase alpha subunit . | gi|94468890 | 34 kDa | 0 | 3 | Mitochondrion | Tricarboxylic acid cycle | Binding/Synthase activity |
| pyruvate dehydrogenase . | gi|157105561 | 39 kDa | 0 | 0 | Mitochondrion | Glycolysis/oxidation reduction | Pyruvate dehydrogenase activity |
| vacuolar H+-ATPase V1 sector subunit D . | gi|94469066 | 28 kDa | 0 | 0 | Mitochondrion | ATP synthesis coupled proton transport | ATPase activity/Protein binding |
| ferrochelatase . | gi|157109844 | 44 kDa | 0 | 0 | Mitochondrion | Heme biosynthetic process | Iron ion binding |
| nipsnap . | gi|157117700 | 32 kDa | 0 | 0 | Mitochondrion | Acetylation | Protein binding |
| isocitrate dehydrogenase . | gi|157134807 | 39 kDa | 0 | 0 | Mitochontrion | Oxidation reduction/Tricarboxylic acid cycle | NAD/NADH binding/Mg2+ binding |
| myosin i . | gi|157130852 | 119 kDa | 5 | 4 | Myosin complex-Cytoskeleton | mRNA transport/protein transport | ATP binding/motor activity |
|  |  |  |  |  |  |  |  |
|  |  |  |  |  |  |  |  |
|  |  |  |  |  |  |  |  |
|  |  |  |  |  |  |  |  |
|  |  |  |  |  |  |  |  |
|  |  |  |  |  |  |  |  |
|  |  |  |  |  |  |  |  |
|  |  |  |  |  |  |  |  |
|  |  |  |  |  |  |  |  |
|  |  |  |  |  |  |  |  |
|  |  |  |  |  |  |  |  |
|  |  |  |  |  |  |  |  |
|  |  |  |  |  |  |  |  |
|  |  |  |  |  |  |  |  |
|  |  |  |  |  |  |  |  |
|  |  |  |  |  |  |  |  |
|  |  |  |  |  |  |  |  |
|  |  |  |  |  |  |  |  |
|  |  |  |  |  |  |  |  |
|  |  |  |  |  |  |  |  |
|  |  |  |  |  |  |  |  |
|  |  |  |  |  |  |  |  |
|  |  |  |  |  |  |  |  |
|  |  |  |  |  |  |  |  |
|  |  |  |  |  |  |  |  |
|  |  |  |  |  |  |  |  |
|  |  |  |  |  |  |  |  |
|  |  |  |  |  |  |  |  |
|  |  |  |  |  |  |  |  |
|  |  |  |  |  |  |  |  |
|  |  |  |  |  |  |  |  |
|  |  |  |  |  |  |  |  |
|  |  |  |  |  |  |  |  |
|  |  |  |  |  |  |  |  |
